# TEVAR for acute type B aortic dissection in pregnant women (35 weeks gestation) with Takayasu's arteritis after cesarean section: a rare case report and literature review

**DOI:** 10.3389/fcvm.2024.1498914

**Published:** 2025-01-17

**Authors:** Taiyu Bi, Xiaotian Duan, Yipeng Yin

**Affiliations:** ^1^ Thoracic Surgery, First Affiliated Hospital of Jilin University, Changchun, Jilin Province, China; ^2^ Nursing Apartment, First Affiliated Hospital of Jilin University, Changchun, Jilin Province, China

**Keywords:** pregnancy, type B aortic dissection, thoracic endovascular aortic repair, Takayasu's arteritis, case report

## Abstract

**Background:**

Takayasu's arteritis (TA) is an autoimmune disease that invades large arteries and mostly occurs in women of childbearing age. It leads to thickening and loss of elasticity of the arterial wall, and eventually vascular occlusion, aneurysm or dissection formation. Type B aortic dissection (TBAD) during pregnancy is a rare disease, which is mostly caused by the increase of blood volume in circulation during pregnancy, the effect of estrogen and progesterone on the aorta, or congenital diseases. TBAD in TA pregnant women is very rare, and the condition is often complicated. It is necessary to make a multidisciplinary treatment plan and determine the timing and method of operation to save the life of mother and fetus.

**Case description:**

We report a pregnant woman at 35 weeks of gestation who presented to the emergency department with sudden and continuously unrelieved chest pain. She had TA for five years. Thoracoabdominal aortic computed tomography with angiography (CTA) showed acute TBAD. Her blood pressure was 209/73 mmHg and could not be lowered with Urapidil, therefore she was diagnosed with complex Stanford type B aortic dissection. She underwent cesarean section under general anesthesia, and the tracheal tube was not removed after surgery. Thoracic endovascular aortic repair (TEVAR) was administered under anesthesia 8 h after cesarean section. Intraoperative aortography showed that the stent blocked the tear of the intima of the aorta, and the false cavity was reduced. Her blood pressure was reduced to the normal range (140/90 mmHg or less), and the baby's vital signs were stable. They were discharged five days later. Use steroids to control TA throughout treatment. One year after the operation, the mother was healthy and the baby developed well.

**Conclusion:**

Early identification and accurate diagnosis should be made for acute TBAD in late pregnancy. Under the premise of stable hemodynamics, the fetus is delivered by cesarean section first and then TEVAR is the preferred treatment. The diagnosis and treatment plan of AD during pregnancy should be developed and implemented by multiple disciplines according to the vital signs of mother and fetus. TA pregnant women should take steroids during pregnancy, closely detect inflammatory indicators, and avoid pathogenic microbial infection, inflammatory state and complications. At the same time, the necessary follow-up is also the key to ensure the treatment effect.

## Introduction

Takayasu arteritis (TA), also known as pulseless disease or arteritis in young women, typically occurs between the ages of 20 and 30 years, with women accounting for 80%–90% of TA cases, with an incidence of 1.2–2.6 per million, and is characterized by nonspecific inflammation of the aorta and its major branches. The pathological changes caused by TA are arterial wall thickening, stenosis, occlusion, calcification or aneurysm formation (1, 2). The incidence of aortic dissection in TA patients is very low, accounting for only 0.87% of TA patients (3).

Aortic dissection (AD) during pregnancy is a rare disease, accounting for 0.1%–0.4% of all AD (4). Type B aortic dissection (TBAD) accounts for 30% of AD during pregnancy (5). 50% of AD during pregnancy occurs in the third trimester (6). Blood enters the arterial wall from the intimal rupture and tears the aortic wall, forming the true and false lumen. Once AD rupture, it will threaten the life of the mother and fetus. TBAD was divided into complex and non-complex types. Complex TBAD accounted for 8.2% of the total TBAD. It is characterized by vascular rupture, rapid aortic dilation, end-organ ischemia, uncontrolled hypertension, and progressive pain (7). Thoracic endovascular aortic repair (TEVAR) is a widely used clinical treatment for complex TBAD (8).

We report a case of acute TBAD in a pregnant woman with TA in the third trimester. TEVAR was performed under anesthesia after cesarean section. To review the cases of TBAD in the third trimester of pregnancy, discuss the etiology, diagnosis and treatment of this disease, and provide evidence for clinicians to diagnose and treat this disease in time.

## Case description

**Figure 1:**
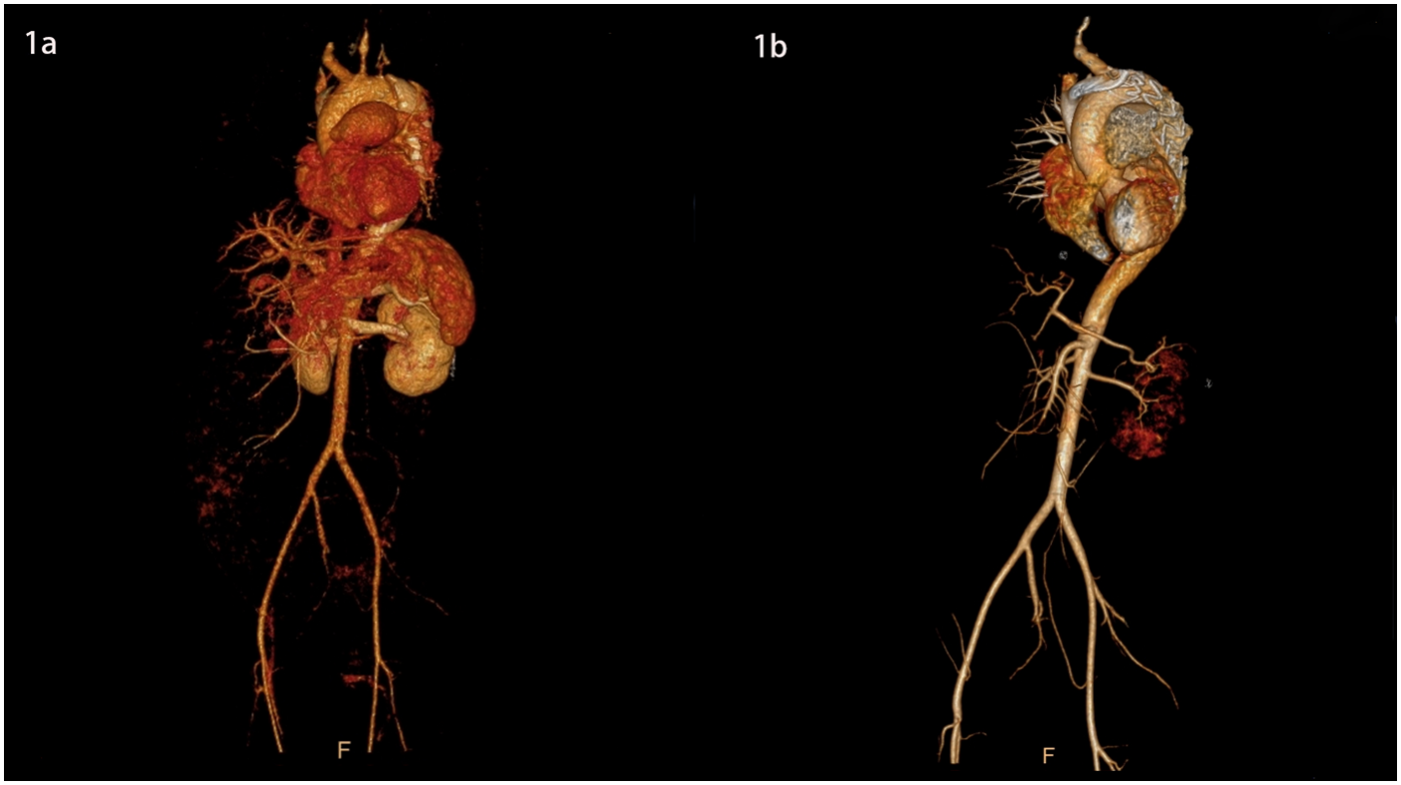
3D images of the thoracic and abdominal aorta before and after TEVAR (front view).

**Figure 2:**
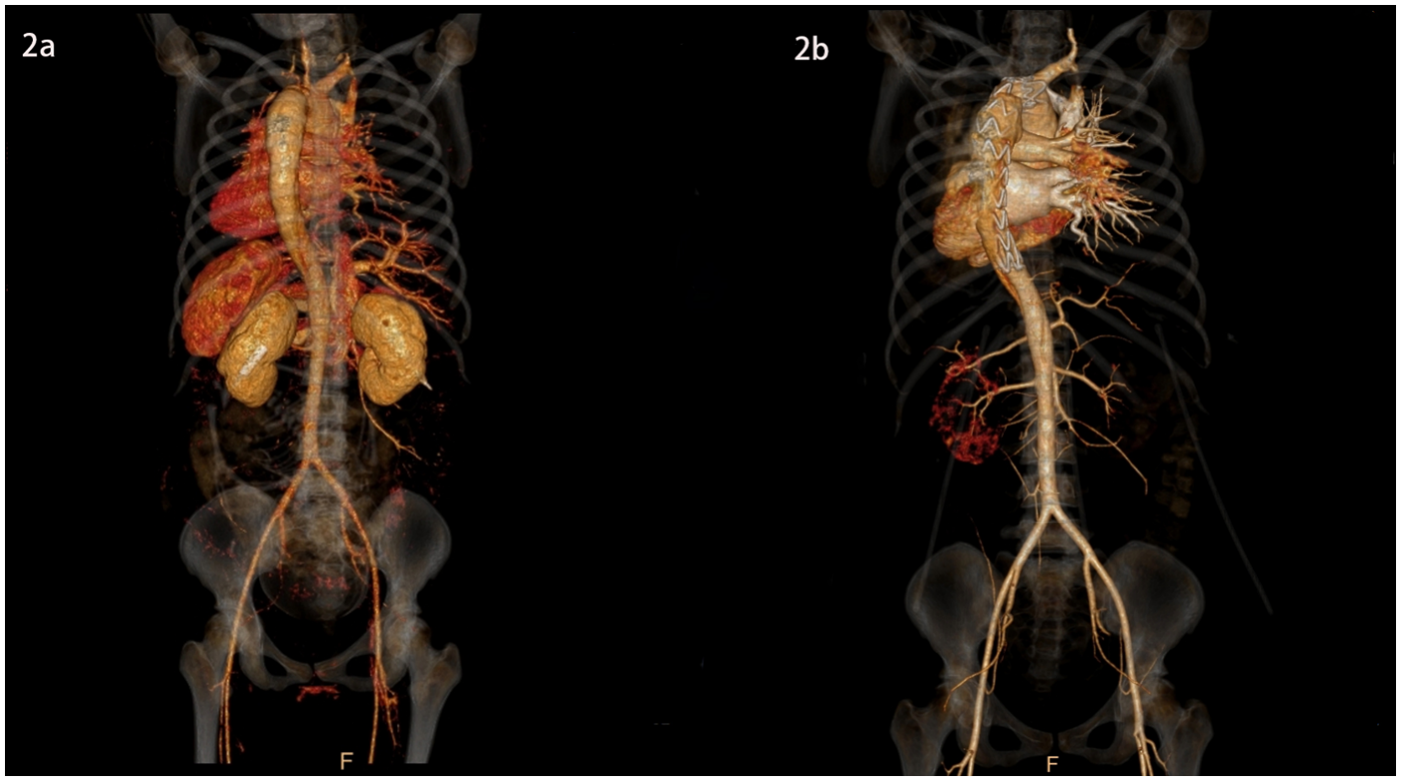
3D images of the thoracic and abdominal aorta before and after TEVAR (rear view).

**Figure 3:**
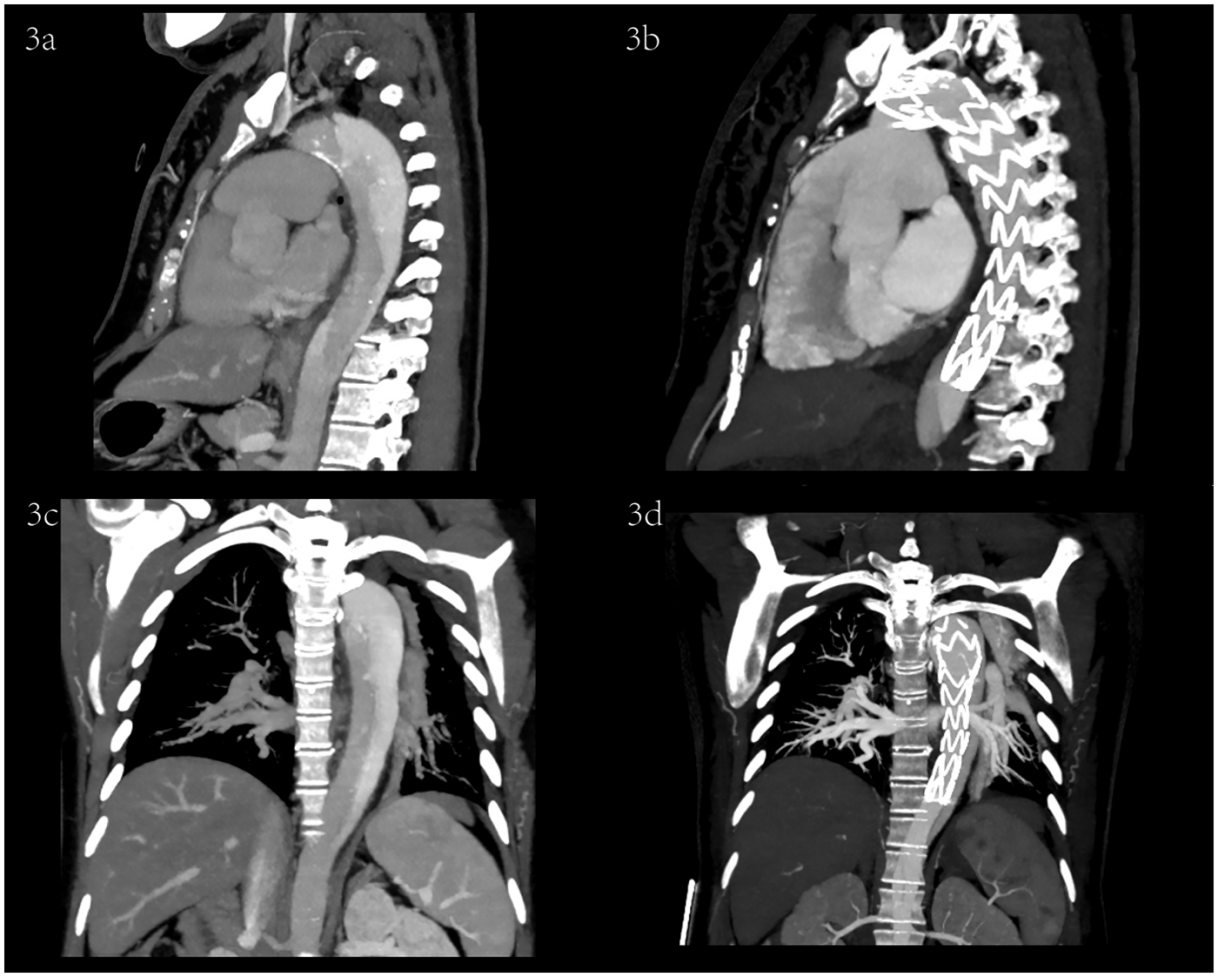
Sagittal and coronal CTA images before and after TEVAR.

A 29-year-old pregnant woman was 157 cm in height and 80 kg in weight. Pregnant at 35 weeks +6 days, gravida 1, para 0. The patient presented to the emergency department with sudden and continuously unrelieved chest pain without a definite cause. Physical examination revealed reduced bilateral carotid pulsation, with the apex pulse located 0.5 cm within the midclavicular line of the fifth intercostal space, regular heart rate on auscultation, no murmur in the auscultation area of each valve, and edema of both lower limbs. Her oxygen saturation was 98%, the blood pressure 131/65 mmHg in the left upper extremity, 165/93 mmHg in the right upper extremity, 200/75 mmHg in the left lower extremity, 209/73 mmHg in the right lower extremity, heart rate 98 beats per minute, and respiratory rate 22 breaths per minute. Emergency echocardiography: EF 64%; Little regurgitation of aortic and pulmonary valve; AD was suspected. Thoracoabdominal aorta CTA: the posterior part of the aortic arch to the descending aorta was double-lumen like, the true lumen was small, the false lumen was large, and the first tear was located in the posterior part of the aortic arch, Acute TBAD (Figures 1a, 2a, 3a,c). Her blood pressure could not be lowered with Urapidil, therefore she was diagnosed with complex Stanford type B aortic dissection. Fetal ultrasound: single fetus, stable vital signs, head presentation, intrauterine pregnancy (gestational age 35 weeks 6 days). D-dimer 1.47 (mg/L), high-sensitivity C-reactive protein 96.75 (mg/dl), white blood cell 15.94  ×  10^9^/L. 7 days before the onset of chest pain, she had an upper respiratory infection, took 2 bags of honeysuckle granules, and had not yet recovered when she was diagnosed with TBAD.

She was taken to hospital 5 years ago due to sudden syncope at work. Arterial ultrasound showed uniform thickening of the intima and media of the bilateral common carotid arteries, and the stenosis degree was 50%–99%; Bilateral external carotid artery stenosis, degree of stenosis 69%–99%; The left internal carotid artery was occluded. The right carotid artery bifurcation stenosis, stenosis degree 70%–99%; The degree of stenosis at the opening of bilateral vertebral arteries was 70%–99%. Stenosis at the origin of bilateral subclavian artery was 70%–99%. Mild stenosis of the left posterior cerebral artery. Pulmonary artery widening; Renal artery was normal. There were no obvious abnormalities in the remaining main vessels. Her erythrocyte sedimentation rate was 22 (mm/h), high-sensitivity C-reactive protein 2.57 (mg/dl), white blood cell 16.69 × 10^9^/L, and hemoglobin 82 (g/L). Her liver and kidney functions were normal. Therefore, she was diagnosed with TA type I (active). She was prescribed oral prednisone therapy at a dose of 60 mg/day, which was tapered. She was also prescribed oral adalimumab and mycophenolate mofetil. Both drugs were discontinued before pregnancy. Before pregnancy, TA was well controlled, without fatigue, syncope, dizziness, headache, urinary system, nervous system symptoms, and stable blood pressure control, about 125–130/80–85 mmHg. During pregnancy, the prednisone dose was 20 mg/day. Her condition for TA was stable until the diagnosis with TBAD.

The treatment plan was developed by a multi-disciplinary team consisting of obstetrics and gynecology, neonatology, cardiac surgery, anesthesiology, rheumatology, and intensive care medicine. Given the stable vital signs of the mother and the fetus, the pregnancy was in the third trimester at 35 + 6 weeks of gestation. She had TA, poor vascular elasticity, multiple arterial stenoses, and a high risk of AD rupture. During the operation, we cooperated closely with cardiac surgery, anesthesiology and neonatology to ensure the safety of the newborn and mother. If the dissection rupture occurred, open vascular replacement would be performed immediately. After the maternal vital signs were stable, TEVAR was performed, and stent was placed to block the gap and reduce the false lumen.

She was immediately treated with medical therapy (pumped with urapidil) to control her blood pressure after admission, and cesarean section was performed 8 h later with a low lower uterine segment. The patient was given endotracheal intubation, low-flow oxygen inhalation, sedation and analgesia, and blood pressure was maintained (≤120/80 mmHg). A transverse 9 cm incision was made 2 cm above the pubic symphysis in the lower abdomen, showing an enlarged uterus with a well-formed lower segment. The lower segment of the uterus was cut transverse for 8 cm. It can be seen that the amount of amniotic fluid is medium and the color is clear. A live baby (Apgar score: 1 min 7, 5 min 9, weight: 2,680 g, length: 46 cm) was delivered by hand, and the umbilical cord was cut off. The baby was in good condition, the skin on the trunk was pink, the head was normal, the heart rhythm was normal, and the limbs could move freely. The baby was given respiratory tract cleaning and warming measures, and then transferred to the neonatal intensive care unit. 20 units of oxytocin were injected into the uterine muscle wall, and 20 units of oxytocin were infused intravenously during the operation. The placenta and fetal membranes were removed by hand. The blood loss was 200 ml. The vital signs were stable during the operation. The patient returned to the cardiac surgery intensive care unit safely after surgery. The blood pressure was stable (130–140/70–90 mmHg), the oxygen saturation was 97%, the respiratory rate was 20–22 breaths per minute, the heart rate was 78–90 beats per minute, and oxytocin 60 units was given for 12 h. The color and volume of lotion were normal. The endotracheal tube was not extubated because of sequential thoracic aortic stenting. Under anesthesia, TEVAR was performed 8 h after cesarean section. Puncture was made through the right femoral artery, and intraoperative aortography confirmed that the tear was about 2 cm lateral to the left subclavian artery, and the contrast agent entered the true and false lumen. A straight stent was placed in the aortic rupture and dissection. Angiography showed that the stent was properly positioned, the false lumen was reduced, the tear was closed, and the fixation was good (Figures 1b, 2b, 3b,d). During the operation, the vital signs were stable, the pressure difference between the two limbs was reduced to 10 mmHg, the dorsalis pedis artery of both lower limbs was clear to touch, and the radial artery of both upper limbs was clear to touch. The patient returned to the cardiac surgery intensive care unit safely after surgery.

She regained consciousness 2 h after surgery and her vital signs were stable. He was transferred out of the cardiac surgical intensive care unit 1 day after surgery. Methylprednisolone 40 mg was administered on day 1 and day 2 after cesarean section. The dose was changed to 20 mg/d after resuming oral diet on the third postoperative day. Her blood pressure was reduced to the normal range (140/90 mmHg or less).On the fifth day after operation, the mother and baby were discharged from the hospital after evaluation by the Department of cardiac surgery and the department of Neonatology. At follow-up 1 year after discharge, the patient was treated with prednisone for TA, her blood pressure was stable, and she had returned to her normal life. The baby weighed 7.8 kg (height 79 cm) and developed well.

## Discussion

Causes of aortic dissection during pregnancy: (1) The amount of blood circulating in the body during pregnancy is 1.5 times greater than that in the non-pregnant state, resulting in increased cardiac output and pressure on the aortic wall. Such physiological changes occur throughout pregnancy. In the third trimester and postpartum, cardiac output increases further compared with the first and second trimester, and the pregnant uterus compresses the aorta. Therefore, the third trimester of pregnancy is a high-risk period for aortic dissection (9); (2) The role of hormones. Estrogen and progesterone inhibit the synthesis of collagen and elastin in the aortic wall and promote the deposition of non-collagen proteins, leading to the degeneration of the aortic wall intima and media (4), and increasing the risk of intimal rupture. (3) congenital factors. Connective tissue disease, Marfan syndrome is the most common one (9), including Ehlers-Danlos syndrome, Loeys-Dietz syndrome, bone aneurysm syndrome and bilobal aortic valve malformation and aortic stenosis, Turner syndrome. These diseases make the aorta vulnerable, and when the aortic root size exceeds a certain threshold (≥40 mm), the risk of aortic dissection increases (10). Braverman et al. reported 29 cases of pregnancy-related aortic dissection, of which 20 (69%) had connective tissue disease (11). Definite family history of aortic aneurysm rupture or dissection, history of hypertension, and congenital heart disease such as aortic coarctation and bicuspid aortic valve are risk factors for aortic dissection during pregnancy (12). (4) Acquired factors. Advanced age and hypertensive disorder complicating pregnancy are risk factors for aortic dissection during pregnancy (13). In most cases, multiple factors act on the aorta of pregnant women to cause dissection. This case had a history of syncope due to Takayasu's arteritis in the active phase, and ultrasound showed multiple arterial stenosis, and the pregnancy was carried out in the stable condition. Although the condition of Takayasu's arteritis was stable and there was no hypertensive disorder of pregnancy, aortic dissection still occurred at 35 gestational weeks. For patients with risk factors for aortic dissection during pregnancy, close attention should be paid to their symptoms and signs during the third trimester and postpartum, and the frequency of prenatal examination should be higher than that of ordinary pregnant women. If necessary, they were admitted to the hospital ahead of time for delivery. For patients with risk factors for aortic dissection during pregnancy, close attention should be paid to their symptoms and signs during the third trimester and postpartum. Connective tissue disease should be fully evaluated by a rheumatologist before pregnancy, and pregnancy should be carried out after the condition is stable. In addition, individualized perinatal medication plan, prenatal examination plan and family management plan were formulated in advance. This requires the cooperation of obstetric specialists. Systolic blood pressure should be controlled below 120–130 mmHg during pregnancy, echocardiography should be performed every 4–12 weeks, and beta-blockers should be used if necessary (13). Arbs and ACEIs are contraindicated. If there are possible symptoms of suspected dissection such as chest pain and syncope, CTA should be performed in time. Asymptomatic dissection requires echocardiography at the time of delivery for diagnosis.

The most common symptom of aortic dissection during pregnancy is sudden severe tearing pain in the chest, back, waist, and abdomen. Some cases also had pain in the head and neck, upper or lower limbs, suggesting involvement of the carotid artery, subclavian artery or left and right iliac arteries. Physical examination revealed a systolic blood pressure difference between the arms greater than 20 mmHg and a weak pulse. Laboratory tests showed an increased white blood cell count and anemia. D-dimer can be used as an early screening index (14). transthoracic echocardiography(TTE), transoesophageal echocardiography(TOE) and electrocardiogram (ECG) were used as screening methods (15).

The most important imaging examination for diagnosis is computed tomography angiography (CTA), including thoracoabdominal aorta CTA and pulmonary artery CTA. Although there is some radiation, CTA can clearly identify the location and classification of the tear, and the length and range of the vessels involved by the dissection. Three-dimensional reconstruction can provide a basis for subsequent treatment. The patient presented with sudden chest pain with a 34 mmHg difference in systolic blood pressure between the arms. The diagnosis was confirmed by CTA of the thoracoabdominal aorta. Pulmonary embolism, spontaneous pneumothorax, amniotic fluid embolism, placental abruption, uterine rupture, and ectopic pregnancy were also excluded. TEVAR has several advantages over open surgery. The aim is to seal the tear, eliminate the false lumen, and direct blood back to the true lumen. The minimally invasive approach avoids major surgery and complications such as bleeding, allows faster postpartum recovery and stability, and has a higher survival compared with open surgery. It also reduces postoperative mortality. In terms of anesthesia, TEVAR reduces the surgical risk associated with cardiopulmonary bypass. In this case, TEVAR was performed under anesthesia 8 h after cesarean section, and the effect was good.

Wang et al. suggested that women with type B dissection in the third trimester should undergo TEVAR prior to delivery. They believe that there is a risk of dissection rupture in the first caesarean section, which may be due to the increased pressure on the aortic wall caused by the increased blood volume returning to the heart after delivery (8). Wang et al. reported seven pregnant patients with acute type A aortic dissection who underwent cesarean section followed by aortic repair after 28 weeks of gestation. In clinical practice, most studies recommend sequential surgery for patients at 28 weeks of gestation and above, that is, cesarean section followed by aortic repair (16, 17). Sumiyoshi et al. reported A pregnant woman with type A aortic dissection at 38 weeks of gestation who underwent cesarean section and sequential aortic repair on the operating table, and the patient survived after surgery (18). Chen et al. reported A case of a pregnant woman with acute type A aortic dissection at 16 weeks of gestation, who underwent aortic arch replacement on the premise of preoperative confirmation of fetal safety. After the operation, the vital signs of the mother and fetus were stable, and the baby was successfully delivered at 38 weeks (19). In this case, a cesarean section was performed at 35 weeks of gestation if the mother and fetus were in stable condition, and there was no postpartum hemorrhage after the cesarean section. TEVAR was performed sequentially under endotracheal intubation anesthesia. We therefore suggest that delivery priority should be the principle of management for aortic dissection in pregnant women with a gestational age of 28–32 weeks. At this time, the fetus is basically mature, and cesarean section can eliminate the hemodynamic pressure brought by pregnancy, better control blood pressure, and eliminate the compression of the pregnant uterus on the large blood vessels. After cesarean section, the vital signs of the pregnant women were stable, and aortic repair was performed (TEVAR should be performed for type B dissection, vascular replacement surgery for type A dissection) (20, 21). Zhu et al. found in 20 pregnant women with aortic dissection that the fetal mortality rate of cesarean section before 28 weeks was 81.8% (9/11), which was much higher than 11% (1/9) after 28 weeks (22). Delivery should not be given priority before 28–32 weeks of gestation, but priority should be given to repair the aorta, stabilize the vital signs of the mother and the fetus, closely monitor the intrauterine condition of the fetus, and continue the pregnancy to term. Type B dissection is treated with medications, strict control of blood pressure, reduction of heart rate, and prevention of continued aortic dilatation, and regular follow-up. Type A aortic dissection can be treated with hypothermic vascular replacement under the safe condition of the fetus, and the pregnancy can be continued to term. For pregnant women at 28–32 weeks, maternal and fetal status should be considered to make intervention decisions. If type A dissection can tolerate surgery, pregnancy should be continued after surgery. Type B dissection can be treated with medical treatment to continue pregnancy or surgery after delivery. If the pregnant woman has confusion, decreased blood pressure, late fetal deceleration, uncontrolled hypertension, aortic root diameter >40 mm and progressive dilatation, rupture of dissection, uterine ischemia and other complications, priority should be given to saving the mother's life, aortic repair surgery and fetal delivery should be performed in time.

TA is a chronic inflammatory vasculitis of unknown etiology, which mainly involves large blood vessels, including the aorta and its main branches, and is more common in women aged 20–30 years (23). Vascular inflammation can lead to the thickening of the arterial wall, the decrease of arterial wall elasticity, the destruction of elastic fibers in the media, the increase of vascular fragility, the fibrosis of the adventitia, and the loss of vascular wall integrity, vascular stenosis, increased blood pressure, thrombosis, aneurysm or dissection formation (24). These lesions are usually asymptomatic. It is usually not diagnosed until definite symptoms develop, many years after the illness. Pregnant women with TA are at high risk for vascular complications, which negatively affect fetal and maternal outcomes. Hypertensive disorders of pregnancy are the most common obstetric complication in TA pregnant women, with an incidence of approximately 24%. Women with active disease are three times more likely to have pathological pregnancies, such as miscarriage, preterm birth, and low birth weight infants, than women with inactive disease. In this case, the patient did not have hypertension and was regularly treated with steroids, but before the onset of the disease, he had a respiratory infection and was in a state of systemic inflammation, which may have precipitated the dissection. Therefore, controlling disease activity before and during pregnancy is essential to optimize maternal and fetal outcomes. We suggest that TA patients need to carry out pregnancy under the premise of stable condition (25). The perinatal blood pressure should be controlled below 120/80 mmHg, and the dosage of steroids and immunosuppressants should be adjusted (26). Steroid therapy and immunosuppressive therapy should be continued during pregnancy to prevent the infection of other pathogenic microorganisms and control the inflammatory state. It is necessary to pay close attention to fetal vital signs and maternal blood pressure during pregnancy, and regularly review white blood cell, ESR, CRP and other inflammatory indicators. Timely prenatal examination can avoid adverse consequences such as dissection rupture, heart failure, renal failure, stroke or maternal death (27, 40–43).

We summarized the age, clinical manifestations, maternal complications, diagnostic methods, extent of aortic dissection invasion, timing and mode of cesarean section surgery, timing and mode of aortic dissection surgery, anesthesia for both procedures, fetal survival and Apgar 1–5 min after birth in patients with acute type B aortic dissection in the third trimester scoring (Table 1). The results were compared with the reported cases. Of the 17 patients, 2 had TA, 4 had MFS, 4 had hypertension, and 12 were confirmed by CTA or CT. All cesarean sections were performed before aortic surgery. Sixteen patients underwent TEVAR and 1 patient underwent open aortic replacement. All mothers and fetuses survived. Therefore, for type B aortic dissection in the third trimester, TEVAR is the correct treatment choice after cesarean section and delivery of the fetus to the department of neonatology.

**Table 1:** Details of 17 pregnant women with acute type B aortic dissection in the third trimester.

Researches	number	Age (years)	Gravidity history	Complications	Clinical manifestation	Diagnosis method	Aortic surgery opportunity	Aortic surgery type of anaesthesia	Aortic surgery	Caesarean section gestational age (weeks)	Caesarean type of anaesthesia	Caesarean operation opportunity	Newborns survival status	Apgar scoring (1–5 min)
Our patient	1	29	G1P0	TA	Chest pain back pain	CTA	After caesarean	General anesthesia	TEVAR	35 + 1	General anesthesia	Before aortic surgery	Survival	7–9
Huang et al. (4)	2	33	G1P0	Gestational hypertension	Back pain radiating to chest	CT	After caesarean	General anesthesia	TEVAR	32 + 3	Spinal anesthesia	Before aortic surgery	Survival	6–8
Price et al. (28)	3	27	G3P2	MFS	Back pain	CTA	After caesarean	General anesthesia	TEVAR	30 + 2	General anesthesia	Before aortic surgery	Survival	–
Wang et al. (13)	4	27	G3P1	Abnormal fetal heart.	Back pain	CTA	After caesarean	General anesthesia	TEVAR	37 + 3	General anesthesia	Before aortic surgery	Survival	–
Ando et al. (3)	5	25	G1P0	TA	Back pain	CT	After caesarean	General anesthesia	TEVAR	35	General anesthesia	Before aortic surgery	Survival	6–9
Patel et al. (29)	6	32	G1P0	MFS	Chest pain radiating to back	MRI	After caesarean	General anesthesia	TEVAR	24	General anesthesia	Before aortic surgery	Survival	–
Stoberock et al. (30)	7	50	–	Gestational hypertension	Back pain	CTA	After caesarean	General anesthesia	TEVAR	32 + 6	Spinal anesthesia	Before aortic surgery	Survival	–
Jayet et al. (31)	8	21	G1P0	TA	Abdominal and back pain	CTA	After caesarean	General anesthesia	Open aortic repair	29	General anesthesia	Before aortic surgery	Survival	–
Hao et al.(32)	9	24	G1P0	NONE	Chest pain radiating to back	MRA	After caesarean	General anesthesia	TEVAR	36	General anesthesia	Before aortic surgery	Survival	9–10
Brener et al.(33)	10	39	G1P0	NONE	Chest pain radiating to back	CTA	After caesarean	General anesthesia	TEVAR	31	General anesthesia	Before aortic surgery	Survival	–
Shu et al. (34)	11	24	G2P0	MFS	Chest pain and dyspnea	CTA	After caesarean	General anesthesia	TEVAR	36 + 5	General anesthesia	Before aortic surgery	Survival	–
	12	22	G1P0	MFS	Chest pain	CTA	After caesarean	General anesthesia	TEVAR	38 + 5	General anesthesia	Before aortic surgery	Survival	–
Kayhan et al. (35)	13	36	G4P3	Hypertension 2 years	Back pain	CT	After caesarean	General anesthesia	TEVAR	30	General anesthesia	Before aortic surgery	Survival	8–10
Katsuragi et al. (36)	14	31	G2P1	NONE	Chest pain	Cardiac ultrasonography	After caesarean	General anesthesia	TEVAR	34	General anesthesia	Before aortic surgery	Survival	–
Rosenberger et al. (37)	15	30	G2P1	Preeclampsia	Chest and abdominal pain	CTA	After caesarean	General anesthesia	TEVAR	30	General anesthesia	Before aortic surgery	Survival	–
Stout et al. (38)	16	25	–	Gestational diabetes	Chest pain and dyspnea	CTA	After caesarean	General anesthesia	TEVAR	26	General anesthesia	Before aortic surgery	Survival	–
Lakhi et al. (39)	17	34	G2P2	TA	Chest pain radiating to the left arm	MRA	After caesarean	General anesthesia	TEVAR	37	General anesthesia	Before aortic surgery	Survival	–

## Conclusions

Acute type B aortic dissection in the third trimester should be recognized early and diagnosed accurately. Blood pressure and heart rate should be actively controlled after diagnosis. Under the premise of hemodynamic stability, cesarean section followed by TEVAR is the preferred treatment. The diagnosis and treatment of aortic dissection during pregnancy should be made according to the vital signs of the mother and fetus and formulated and implemented by a multidisciplinary team. TA pregnant women should take steroids during pregnancy, closely detect inflammatory markers, avoid pathogenic microorganism infection and inflammatory state, and avoid complications. At the same time, the necessary follow-up is also the key to ensure the treatment effect.

## Data Availability

The original contributions presented in the study are included in the article/Supplementary Material, further inquiries can be directed to the corresponding author.
